# Community Risk Factors for Ocular Chlamydia Infection in Niger: Pre-Treatment Results from a Cluster-Randomized Trachoma Trial

**DOI:** 10.1371/journal.pntd.0001586

**Published:** 2012-04-24

**Authors:** Abdou Amza, Boubacar Kadri, Baido Nassirou, Nicole E. Stoller, Sun N. Yu, Zhaoxia Zhou, Stephanie Chin, Sheila K. West, Robin L. Bailey, David C. W. Mabey, Jeremy D. Keenan, Travis C. Porco, Thomas M. Lietman, Bruce D. Gaynor, for the PRET Partnership

**Affiliations:** 1 Programme National de Lutte Contre la Cecité, Niamey, Niger; 2 F.I. Proctor Foundation, University of California San Francisco, San Francisco, California, United States of America; 3 Dana Center for Preventive Ophthalmology, Wilmer Eye Institute, Johns Hopkins University, Baltimore, Maryland, United States of America; 4 Clinical Research Unit, Department of Infectious and Tropical Diseases, London School of Hygiene and Tropical Medicine, London, United Kingdom; 5 Department of Ophthalmology, University of California San Francisco, San Francisco, California, United States of America; 6 Department of Epidemiology and Biostatistics, University of California San Francisco, San Francisco, California, United States of America; 7 Institute for Global Health, University of California San Francisco, San Francisco, California, United States of America; 8 Center for Infectious Disease and Emergency Readiness, University of California, Berkeley, California, United States of America; University of Cambridge, United Kingdom

## Abstract

**Background:**

Trachoma control programs utilize mass azithromycin distributions to treat ocular *Chlamydia trachomatis* as part of an effort to eliminate this disease world-wide. But it remains unclear what the community-level risk factors are for infection.

**Methods:**

This cluster-randomized, controlled trial entered 48 randomly selected communities in a 2×2 factorial design evaluating the effect of different treatment frequencies and treatment coverage levels. A pretreatment census and examination established the prevalence of risk factors for clinical trachoma and ocular chlamydia infection including years of education of household head, distance to primary water source, presence of household latrine, and facial cleanliness (ocular discharge, nasal discharge, and presence of facial flies). Univariate and multivariate associations were tested using linear regression and Bayes model averaging.

**Findings:**

There were a total of 24,536 participants (4,484 children aged 0–5 years) in 6,235 households in the study. Before treatment in May to July 2010, the community-level prevalence of active trachoma (TF or TI utilizing the World Health Organization [WHO] grading system) was 26.0% (95% CI: 21.9% to 30.0%) and the mean community-level prevalence of chlamydia infection by Amplicor PCR was 20.7% (95% CI: 16.5% to 24.9%) in children aged 0–5 years. Univariate analysis showed that nasal discharge (0.29, 95% CI: 0.04 to 0.54; *P* = 0.03), presence of flies on the face (0.40, 95% CI: 0.17 to 0.64; *P* = 0.001), and years of formal education completed by the head of household (0.07, 95% CI: 0.07 to 0.13; *P* = 0.03) were independent risk factors for chlamydia infection. In multivariate analysis, facial flies (0.26, 95% CI: 0.02 to 0.49; *P* = 0.03) and years of formal education completed by the head of household (0.06, 95% CI: 0.008 to 0.11; *P* = 0.02) were associated risk factors for ocular chlamydial infection.

**Interpretation:**

We have found that the presence of facial flies and years of education of the head of the household are risk factors for chlamydia infection when the analysis is done at the community level.

**Trial Registration:**

ClinicalTrials.gov NCT00792922

## Author Summary

Trachoma is one of the most important neglected tropical diseases because it is the leading cause of blindness from an infection in the world. There are about 1.3 million persons blind from the disease and many more at risk of blindness in the future. It is caused by the common bacterium *Chlamydia trachomatis* and can be treated with mass drug administrations (MDA) of azithromycin. We have begun a clinical trial in Niger, a country with limited resources in Africa, to determine the best treatment strategy. Our study from May to July 2010, which began before MDA's were given, showed that 26% of children aged 0–5 years were infected with the disease. In these children, we found that discharge from the nose, presence of flies on the face, and the number of years of education completed by the head of the household were risk factors for infection in 48 different communities. We hope to use this information about risk factors of infection to help guide future studies for trachoma and also to help with the WHO goal of eliminating the disease worldwide by the year 2020.

## Introduction

### Background

Trachoma is an ocular infection caused by *Chlamydia trachomatis*. The World Health Organization (WHO) has implemented treatment guidelines that include mass antibiotic administration, facial cleanliness campaigns and environmental improvements in an effort to control and ultimately eliminate the disease [1]. The risk factors for infection are poorly characterized on a community level. This study establishes a knowledge base that assesses these community-level risk factors in an effort to guide the WHO and other programmatic efforts. Some of the risk factors thought to be predictive of trachoma include facial cleanliness (nasal or ocular discharge and presence of flies on the face), access to a latrine, access to clean water, and education. Many studies have identified individual-level risk factors, such as hygiene and latrine use [2], [3], but trachoma is a communicable disease with community-level risk factors and treatment. Individuals can be affected by their neighbors, regardless of whether they themselves are compliant with their prescribed antibiotic treatment or adherent with other recommended environmental changes. Here, we analyze community-level risk factors for ocular chlamydia infection in Niger in an effort to improve trachoma control and elimination strategies. In this arm of the Partnership for the Rapid Elimination of Trachoma (PRET) trial [4], [5], we assess 48 communities before treatment and determine the importance of several community-level risk factors for ocular chlamydial infection.

## Methods

### Study Setting and Design

Niger, in the Sahel region of West Africa, is one of the poorest and least developed countries in the world, ranking in the bottom 1% of countries in the 2010 United Nations Human Development Index [6]. Trachoma is endemic within Niger's population of roughly 14 million [7], with estimated regional prevalences of between 5% and 49% in children aged 1–9 years [8]. Our study methods have been previously described [4] and are briefly summarized below.

### Site Selection

In Niger, contiguous administrative units are known as *grappes*, and are referred to as *communities* in this manuscript. A community is the smallest population unit for which health services are organized and within which trachoma programs are implemented. The study took place in the Matameye district in the Zinder region of Niger. Communities were selected from among 6 health centers (Centre de Santé Intégrée or CSIs) and were eligible for inclusion if they had an estimated total population of between 250 to 600 persons, generally encompassing between 50 and 100 children in the eligible age range for treatment. Other community inclusion criteria were distance >4 kilometers from the center of any semi-urban area (communities which are close to an urban center are believed to have a lower prevalence of trachoma), and prevalence of active trachoma (TF and/or TI)≥10% in children aged 0–5 years. There were a total of 235 eligible communities in the 6 CSIs of which 72 (31%) satisfied the inclusion criteria for community size and 48 of these were selected for inclusion in the study.

### Community Randomization Arms

**Figure 1:**
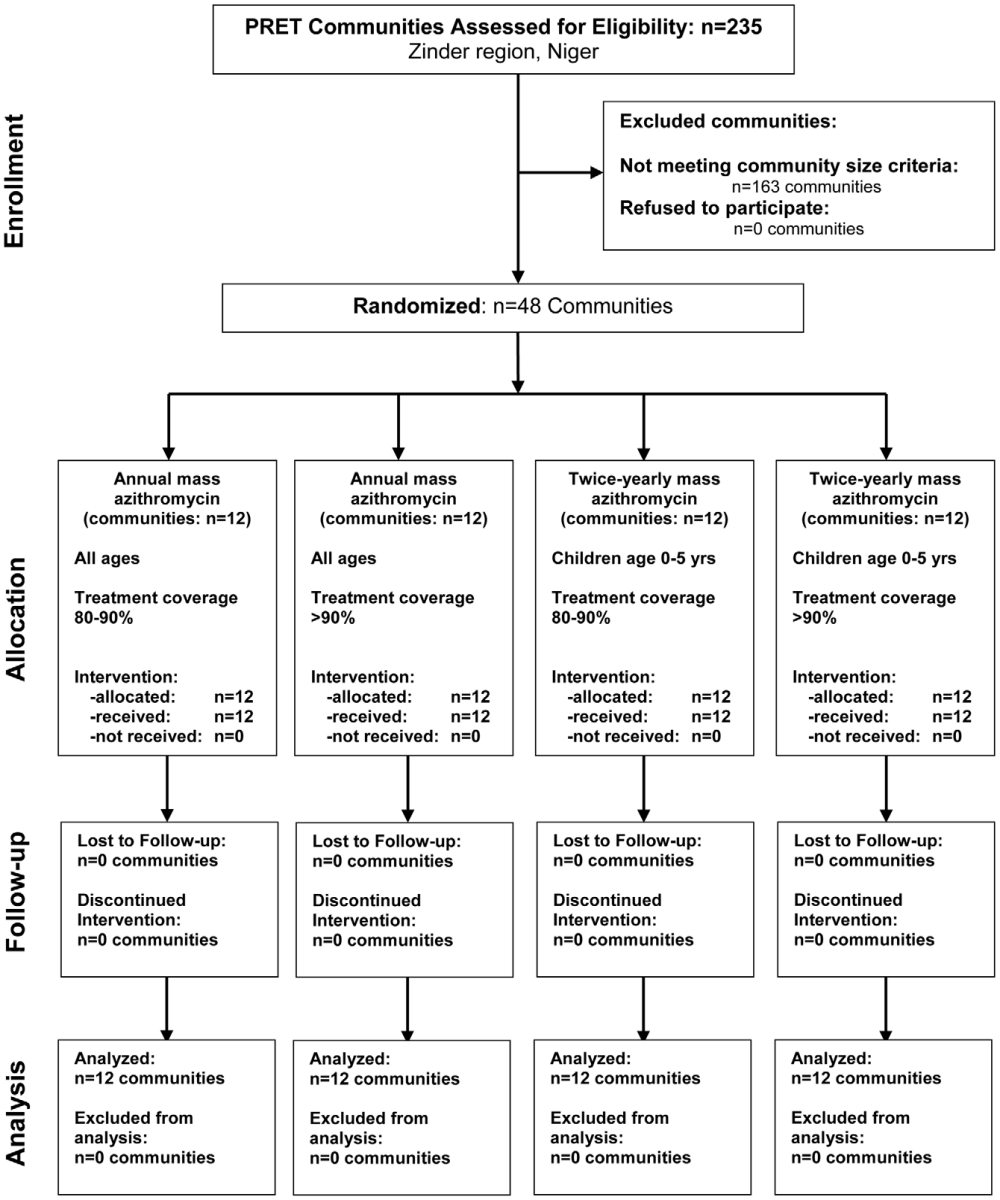
Consort flow diagram: cluster-randomized trachoma trial in Niger.

In a 2×2 factorial design, 48 communities were randomly allocated into 4 treatment arms with 12 communities in each arm (Figure 1). Randomization of communities and sentinel individuals to the treatment arms was done using RANDOM and SORT functions in Excel (Version 2003) by TP and BN. Note that only pretreatment results are presented here.

### Communities and Sentinel Children

To determine the impact of mass antibiotic administration on clinical trachoma and ocular chlamydia infection, a random sample of 50 to 100 children aged 0 to 5 years was established as the sentinel group for the study in all enrolled communities prior to treatment. No adjustments were made for missing individuals from the census and all analyses were performed at the community level on an intention-to-treat basis.

### Data Collection

Baseline data were collected on the following measures: trachoma clinical grade, facial cleanliness, ocular swabs, and ocular photographs. Grader validation was done in a 2-step process: in the first step, research leaders attended a workshop conducted in February 2008 in Ethiopia where trachoma is hyper-endemic, to standardize methods for the trial. Certification of researchers for trachoma grading required a chance corrected agreement (kappa statistic ≥0.6) with an experienced grader (RB) over the scoring signs of clinically active trachoma (TF and/or TI in the WHO system) in validation exercises in both the classroom (photographic collection) and the field. In the second step, clinical graders in Niger were eligible to perform ocular grading for the trial if they had attained a chance corrected agreement (kappa statistic ≥0.6) with a certified grader over the scoring signs of clinically active trachoma. The pretreatment visit for this trial was conducted in Niger from May to July 2010, where 3 ophthalmic nurses (TSO's, or Technicien Superior en Ophtalmologie) received a kappa score on photo grading validation of 0.96, 1.00 and 0.88 (against senior grader RB).

### Census

A population census of all 48 study communities was conducted by trained personnel, masked to the treatment arm, prior to the baseline visit. Study personnel were also masked to the prevalence of clinical trachoma and ocular chlamydia infection. The census team moved from house to house, enumerating all residents in all households, collecting baseline characteristics information. The 100% census allowed us to create a sampling frame from which we could randomly select sentinel children with equal probabilities.

### Examination

After obtaining consent, conjunctival examination for trachoma and conjunctival swabbing for chlamydial PCR were performed on all sentinel children [9], [10]. Clinical grading of the right everted superior tarsal conjunctiva was performed using a 2.5× magnifying loupe and adequate sunlight or a torch light according to the WHO simplified grading system [11]. Prior to conjunctival swabbing, a trained photographer took a minimum of 2 photographs of the right eyelid of all child participants using a Nikon D-series camera and a Micro Nikon 105 mm; f/2.8 lens (Nikon, Tokyo, Japan).

After conjunctival examination, a Dacron swab was passed firmly 3 times over the right upper tarsal conjunctiva, rotating 120 degrees between each pass [9], [10]. To assess for field contamination, negative field control swabs were collected from 5 randomly selected children in each community. The negative field controls were collected immediately after the ocular swabs were collected; a new swab was passed within 5 cm of, but not contacting, the participant's exposed conjunctiva [9]. Examiners changed gloves before examining each new participant. All of the samples were placed immediately at 4°C in the field and frozen at −20°C within 10 hours. Swabs were shipped at 4°C to University of California, San Francisco, CA, USA, where they were stored at −80°C until processing [12]. The Amplicor PCR assay (Roche Diagnostics, Branchburg, NJ, USA) was used to detect *C. trachomatis* DNA and samples were pooled for processing to save time and cost, as previously described [13]. For laboratory standardization and validation between PRET study sites, a set of 20 samples (both positive and negative) was sent to a laboratory at Johns Hopkins University (Baltimore, MD, USA) for processing. Results were considered valid only when agreement between labs was at least 90%.

### Risk Factors

During the census, community-level data were collected on household characteristics, including number of years of education completed by head of household; distance to the primary water source >30 min walk; presence of a latrine; and awareness of a programmatic face-washing campaign in the community within the previous year. Facial cleanliness was measured for each child who presented for an exam, including the presence of ocular discharge (on the eyelashes or eyelids), nasal discharge (on nares, cheeks, or lips), and presence of flies on face (presence of ≥1 fly on the face while observing for 3 seconds).

### Statistical Methods

Data were entered into a database (MS Access v2007) developed at the Dana Center, Johns Hopkins University as previously described [5]. Data were double-entered by different data entry clerks and discrepancies were resolved by reference to the original forms. Queries of all data discrepancies were identified using MS Access prior to any analysis. The intention-to-treat statistical analysis was performed using the statistical package R (http://www.r-project.org), 2.12 for MacIntosh. All pretreatment characteristics were summarized at the community level. Univariate associations with ocular chlamydia infection in children aged 0–5 years were tested using linear regression, with permutation-based significance testing because of non-normality. Multivariate regression was conducted using demographic and clinical (non-trachoma) predictors by selecting the best univariate predictors (no more than 4 included predictors, because the number of communities was only 48). Because stepwise regression may result in overfitting [14], we used Bayes model averaging based on the Bayes information criterion to conduct multiple regression (R package BMA, v. 3.14) [15]. This technique yields estimates for each regression coefficient and a posterior probability that each differs from zero (posterior effect probabilities). Because of heteroskedasticity of the outcome variable, we reported confidence intervals derived from bootstrap resampling of the residuals following ordinary linear regression [16] (with 10,000 replications) for each regression coefficient.

### Human Participants and Consent Procedures

Ethical approval for this study was obtained from the Committee for Human Research of the University of California, San Francisco and le Comite Consultatif National d'Ethique du Ministere de la Sante Publique, Niger (Ethical Committee, Niger Ministry of Health). Oral consent was obtained from the village leaders, and written (thumbprint) consent from the study participant (or the child's parent or guardian) at the time of examination. The study was carried out in accordance with the Declaration of Helsinki. An independent data and safety monitoring committee appointed by the PRET study executive committee oversaw the design and implementation of the study and performed annual reviews of quality assurance and adverse events.

## Results

### Overview of Communities and Households

The 48 randomly selected communities included 24,536 total individuals in 6,235 households (Table 1). Exams were conducted and ocular swabs collected from a total of 4,484 children aged 0–5 years from May to July 2010. The mean prevalence of clinical trachoma (TF) was 26.0% (95% CI: 21.9% to 30.0%) and the mean prevalence of ocular *C. trachomatis* infection was 20.7% (95% CI: 16.5% to 24.9%) in the 48 communities (Table 2). The community-level prevalence of infection and the community-level prevalence of TF were highly correlated (Pearson correlation 0.63, 95% CI: 0.42 to 0.78; *P*<0.001).

**Table 1:** Baseline characteristics of cluster-randomized trial in Niger.

Characteristic	
Communities	48
Total population	24,536
Households	6,235
Children in study^1^	4,484
Persons/household, median (IQR)	7 (6)
Households >30 mins to water	355; 5.7%, (4.9% to 6.7%)
Households with latrine access	667; 10.7%, (9.6% to 11.9%)
Community prevalence children^1^	28.2% (27.6% to 28.8%)
Community prevalence male children^1^	49.5% (48.3% to 50.1%)

95% confidence intervals in parentheses, unless otherwise noted.

1 Aged 0–5 years.

**Table 2:** Characteristics in children aged 0–5 years in 48 communities in Niger.

Characteristic	
Mean prevalence TF^1^	26.0% (21.9% to 30.0%)
Mean prevalence TF/TI^2^	28.3% (24.1% to 32.4%)
Mean prevalence *C. trachomatis* 3	20.7% (16.5% to 24.9%)
Pearson correlation between TF & infection	0.63 (0.42 to 0.78), *P*<0.001
Pearson correlation between TF/TI & infection	0.71 (0.53 to 0.83), *P*<0.001

All values with 95% confidence intervals in parentheses.

1 TF = trachomatous inflammation, follicular.

2 TI = trachomatous inflammation, intense.

3 *C. trachomatis* by Amplicor PCR.

### Field Controls and Laboratory Standardization

A total of 238 negative field control swabs were collected; 12 (5.0%) were PCR positive for chlamydia. As part of a quality control investigation, it was determined that 11 of these 12 (91.7%) field controls were collected by a single examiner and steps were immediately taken to improve the field training for specimen collection in all field workers to reduce contamination. All positive field controls had corresponding positive ocular swabs. The pre-specified level of agreement between laboratories at UCSF and Johns Hopkins University was maintained at >90%.

### Risk Factors for Ocular Chlamydia Infection - Univariate Analysis

In the univariate model (Table 3) using simple linear regression, there was increased risk of ocular chlamydia infection in communities where children had nasal discharge (0.29, 95% CI: 0.04 to 0.54; *P* = 0.03) or flies on the face (0.40, 95% CI: 0.17 to 0.64; *P* = 0.001). Other significant predictors of chlamydia infection were years of education of the head of household (0.07, 95% CI: 0.007 to 0.13; *P* = 0.03), proportion of children aged 0 to 1 year (−0.96, 95% CI: −1.73 to −0.19; *P* = 0.02) and prevalence of clinical trachoma TF (0.65, 95% CI: 0.42 to 0.88; *P*<0.001) (Table 3).

**Table 3:** Univariate analysis of chlamydia infection predictors in children 0–5 years in 48 communities in Niger.

Prevalence of characteristic	Coefficient	R-squared	*P*-value
Proportion female	0.44 (−0.51 to 1.40)	0.02	0.38
Proportion 0 or 1 years	−0.96 (−1.73 to −0.19)	0.11	0.02
Ocular Discharge^1^	0.19 (−0.03 to 0.41)	0.06	0.10
Nasal Discharge^2^	0.29 (0.04 to 0.54)	0.10	0.03
Flies on face^3^	0.40 (0.17 to 0.64)	0.20	0.001
Mean no. of persons/household	0.003 (−0.01 to 0.02)	0.003	0.73
Mean no.years of education completed by head of household	0.07 (0.007 to 0.13)	0.09	0.03
Time to water source >30 min walk	−0.03 (−0.29 to 0.23)	0.001	0.85
Latrine presence	0.13 (−0.19 to 0.45)	0.01	0.43
Number of households (1000)	0.29 (−0.99 to 1.58)	0.004	0.66
Education program	0.10 (−0.05 to 0.25)	0.04	0.20
Prevalence TF	0.65 (0.42 to 0.88)	0.40	P<0.001
Prevalence TI	1.61 (1.19 to 2.03)	0.55	P<0.001
Prevalence TF or TI	0.71 (0.51 to 0.92)	0.50	P<0.001

1 On eyelashes or eyelids.

2 On nares, cheeks or lips.

3 Presence of ≥1 fly on the face during a 3-second examination.

### Risk Factors for Ocular Chlamydia Infection - Multivariate Analysis

In the multivariate analysis using Bayes model averaging (Table 4), among the measured risk factors, chlamydia infection in communities was associated only with flies on face (0.26, 95% CI: 0.02 to 0.49, *P* = 0.03) and level of education of head of household (0.06, 95% CI: 0.008 to 0.11, *P* = 0.02). Other characteristics, including gender, ocular discharge, nasal discharge, number of individuals per household, water access >30 mins, and latrine access, were not significant risk factors in this model. Ordinary linear regression gave confidence intervals which were slightly wider than those derived from bootstrap resampling.

**Table 4:** Multivariate analysis of chlamydia infection predictors in children 0–5 years in 48 communities in Niger.

Prevalence of characteristic	Coefficient	*P*-value	Posterior probability (%)
Fraction of 0–5 year olds less than 1 year	−0.72 (−1.42 to −0.02)	0.05	52.9
Nasal Discharge^1^	0.11 (−0.12 to 0.35)	0.35	23.8
Flies on face^2^	0.26 (0.02 to 0.49)	0.03	85.5
Mean no. years education completed by head of household	0.06 (0.008 to 0.11)	0.02	55.4

All characteristics are proportions at the community level using Bayes model averaging.

1 On nares, cheeks or lips.

2 Presence of ≥1 fly on the face during a 3-second examination.

## Discussion

We have found that communities with higher percentages of younger children, nasal discharge, facial flies, and number of years of education of the head of the household are associated with higher community prevalence of chlamydia infection in univariate analysis. Community-level facial flies and years of education of head of household are significantly associated with the prevalence of chlamydia infection in multivariate analysis. Flies are thought to be important in trachoma transmission but studies have been done only on the individual level [2], [5], [17]–[19] or have used clinical outcomes rather than more objective laboratory measurements [2], [17], [18]. Chlamydia DNA has been found on 15% of flies in areas hyperendemic for trachoma [20] and so flies are believed to be vectors for the spread of chlamydia in endemic areas [21]. Our study is the first to show the association between community-level fly density flies and community-level ocular chlamydia infection.

Other studies performed on the individual-level have concluded that general education is associated with less trachoma [5], [22]–[25], but our study in Niger found the opposite and chlamydia infection was correlated directly with the self- reported years of education of the head of the household. Trachoma is felt to be disease which aggregates among the poor and uneducated [26], and studies have shown health education can improve trachoma control [27]. However, a study in Mali showed the odds of trachoma was higher in households where children attended a traditional school compared to households where children attended a modern school. Mali shares a border with Niger of greater than 860 km and is likely more similar to Niger than more distant areas. The structure of the schools and the way in which children congregate in these schools is just as important as the years of education that have been received.

We have used community-level predictors and community-level outcomes because trachoma is a communicable disease; poor hygiene and specific behavior of others in the same household and neighborhood may increase the risk of infection in the entire community [26], [28]–[30]. For these reasons the WHO strategy for reducing trachoma is implemented at the community level with community-wide interventions. Other trachoma studies have been cluster-randomized but then analyzed at the level of the individual [10], [27], [31]. Like our study in Niger, the Tanzania arm of the PRET study found that facial flies are associated with ocular chlamydia infection [5]. However, this individual-level study also showed that facial cleanliness is a risk for ocular chlamydia infection, an association which we were unable to identify. Reinfection is known to occur at the community level and an individual-level approach does not take this into account. In our study, we look for risk factors for higher community prevalence of ocular chlamydia infection.

The clinical exam for trachoma has been shown to be unreliable and poorly correlated with infection in some situations [32]–[34]. We chose to use the more objective, masked, outcome of ocular chlamydia infection by PCR in our study. Note that we did include the clinical exam in the multivariate predictor model of infection by design, because TF and TI are *consequences* of infection rather than *causes* of it. However, in a different context, programs that have access to clinical surveys may be interested in the associated level of infection that we have measured here (Table 5). It is important to keep in mind the clinical exam for trachoma (TF) is close to 90% sensitive but only 30% specific in latent class analysis, leading to overtreatment in some situations [35]. Furthermore, the poor correlation between the clinical signs of trachoma and the laboratory evidence of infection with *C. trachomatis*, becomes more problematic as the community-level of infection decreases following mass treatment [36]. Nevertheless, the clinical exam is inexpensive and easy to perform. It will remain an important tool for the WHO in their treatment guidelines and continued efforts to understand the relationship between clinical trachoma and infection with the causative bacteria is critical.

**Table 5:** Multivariate analysis of clinical trachoma as a predictor of chlamydia infection in children 0–5 years.

Prevalence of characteristic	Coefficient	*P*-value
Prevalence TF^1^	0.31 (0.08 to 0.54)	0.01
Prevalence TI^2^	1.23 (0.75 to 1.71)	<0.001

1 TF = trachomatous inflammation, follicular.

2 TI = trachomatous inflammation intense.

There are several limitations to this study. First, we evaluated only a sample of sentinel children in the communities rather than all individuals and this may have produced bias in our estimates. Note that laboratory workers were masked to the identities of the sentinel children chosen and the communities in which they lived. Second, although all of the selected communities for inclusion in the study were evaluated successfully as planned, some individuals who were randomly selected for inclusion were missing; if individuals were not missing at random, this could also have created bias. All analyses were done on an intention-to-treat basis with no adjustments for missing individuals. Third, the evidence for risk factors of infectious ocular chlamydia that we have found in rural Niger may not be generalizable to other trachoma-endemic areas in other countries and continents because of differences in important, unmeasured cultural or environmental characteristics. The finding that more years of education of the head of household is associated with *higher* levels of chlamydia infection is counterintuitive. The study questionnaire asked specifically for the number of years of formal education that were completed. Religious education, a form of learning that is very common in this area, was not included in this estimate. If the education of adults is by methods other than formal education in households, our measurement could have been an underestimate of the total amount of education interfering with our ability to capture an association with infection.

In summary, we have found that facial flies and years of education of the head of the household are associated with community-level prevalence of ocular chlamydia infection. Further analyses will be performed as treatment begins in the study and continues over the next 3 years.

## Supporting Information

Checklist S1 **CONSORT Checklist.**
(DOC)

Protocol S1 **Trial Protocol.**
(PDF)
